# Photosynthetic Traits and Nitrogen Uptake in Crops: Which Is the Role of Arbuscular Mycorrhizal Fungi?

**DOI:** 10.3390/plants9091105

**Published:** 2020-08-27

**Authors:** Raffaella Balestrini, Cecilia Brunetti, Walter Chitarra, Luca Nerva

**Affiliations:** 1 National Research Council-Institute for Sustainable Plant Protection (CNR-IPSP), 10125 Turin, Italy; cecilia.brunetti@ipsp.cnr.it (C.B.); walter.chitarra@crea.gov.it (W.C.); luca.nerva@crea.gov.it (L.N.); 2 Council for Agricultural Research and Economics, Research Center for Viticulture and Enology, (CREA-VE), 31015 Conegliano (TV), Italy

**Keywords:** symbiosis, plant performance, nutrient use efficiency

## Abstract

Arbuscular mycorrhizal (AM) fungi are root symbionts that provide mineral nutrients to the host plant in exchange for carbon compounds. AM fungi positively affect several aspects of plant life, improving nutrition and leading to a better growth, stress tolerance, and disease resistance and they interact with most crop plants such as cereals, horticultural species, and fruit trees. For this reason, they receive expanding attention for the potential use in sustainable and climate-smart agriculture context. Although several positive effects have been reported on photosynthetic traits in host plants, showing improved performances under abiotic stresses such as drought, salinity and extreme temperature, the involved mechanisms are still to be fully discovered. In this review, some controversy aspects related to AM symbiosis and photosynthesis performances will be discussed, with a specific focus on nitrogen acquisition-mediated by AM fungi.

## 1. Introduction

An enhanced photosynthetic efficiency might help achieve the sustainable yield increases required to meet future food and energy demands, mainly considering the climate estimations for the coming decades, with increased temperatures, drought and soil salinization that are also correlated to soil degradation [1,2]. This scenario requests for more stress-tolerant and climate-flexible crops [3,4]. It is in fact already known that plant photosynthetic efficiency is closely related to growth and development traits [5]. The plant root system provides a unique ecological niche for soil microbiota that colonize the rhizosphere, roots and to a certain extent above ground parts. This narrow layer of soil, which is under the direct influence of plant roots, is considered one of the most complex ecosystems and a hot spot for microbial activities [6]. Arbuscular mycorrhizal (AM) fungi are one of the most important groups of plant symbionts and positively affect several aspects of plant life, i.e., improved nutrition, better growth, stress tolerance, and disease resistance [7]. These fungi, which are obligate symbionts, provide mineral nutrients to plants in exchange for carbon compounds (carbohydrates and lipids). Although substantial amounts of resources are exchanged, the factors that regulate trade in the AM symbiosis are poorly understood [8,9]. It has been suggested that an increased photosynthetic rate in leaves of mycorrhizal plants might be due to enhanced rhizospheric sink strength or to a mycorrhizal-dependent increase in the P status of AM plants grown at low P [10]. Increased total CO_2_ assimilation can be in fact linked to an enhanced plant growth due to an improved nutrition, resulting in a higher concentration of chlorophyll, photosynthetic enzymes, ATP, and inorganic P (Pi) in leaves that could stimulate rates of photosynthesis. However, the fact that photosynthesis is also regulated by the source-sink relations of the plant should be keep in consideration. Quoting Jansson et al., 2018, *photosynthetic uptake of carbon in plants occurs in source tissues which are net exporters of photosynthate, such as mature leaves. Excess photosynthate, mainly in the form of sucrose, moves from mesophyll cells to the phloem, where it is transported to sink tissues, i.e., net importers of photosynthate, such as roots, seeds, stems, or young leaves, where it is metabolized and/or stored*. It is worth noting that up to 20% of photosynthates has been reported to be released to the rhizosphere, through exudation but also as secretions and root epidermal cells [3]. The role of AM symbiosis as carbon sinks for plants has been already reported [11,12,13], suggesting a role for this group of fungi in increasing rhizospheric sink strength, and thus enhancing photosynthesis in source leaves. However, it was recently reported that substantial amounts of photosynthetically fixed C did not allocated to fungal symbionts supplementing the soil with a commercial AM fungal inoculum [14]. Despite these contrasting data, looking at transcriptomics and proteomics data from leaves of AM plants, the up-regulation of genes and proteins involved in photosynthesis and related processes has been reported in wheat [15]. Particularly, genes and proteins related to RuBisCO large subunit-binding protein, Photosystem II 10 kDa polypeptide, Sucrose synthases and a cell wall invertase, involved in the sucrose cleavage yielding UDP-glucose and fructose, have been found to be induced by AM symbiosis [15]. Moreover, proteomics highlighted the accumulation of a ferrochelatase-2 (FC2), producing heme for the photosynthetic machinery, two delta-aminolevulinic acid dehydratases that are implicated in chlorophyll biosynthesis, in addition to proteins involved in photosystem II repair such as two peptidyl-prolyl cis-trans isomerases (PPIases) and a Protease Do-like 5 [15]. Additionally, three genes belonging to the photosynthesis category (i.e., two genes with a role in the light reactions and one gene related to Calvin cycle) have been found to be mycorrhizal-responsive in tomato leaves [16]. Recently, physiological and transcriptomic data showed that AM symbiosis attenuated the reduction of photosynthetic CO_2_ assimilation rate and the downregulation of photosynthesis-related genes due to cucumber mosaic virus infection [17]. However, different studies that investigate AM symbiosis and plant performance are mainly related to the improvement in tolerance to abiotic stresses [18,19]. Recently, Li et al. [20] showed through transcriptomics that 24 differentially expressed genes (DEGs) related to photosynthesis and respiratory metabolism were regulated during symbiosis upon low temperature stress, providing new findings into low temperature tolerance mediated by AM fungi. In detail, AM root colonization had positive effects on low temperature tolerance, impacting the expression of genes correlated to light harvest complex (LHC) and photosystems PS I and PS II. Overall results suggested a PS I and PS II photoinhibition alleviation coupled to a decrease in ROS production and accumulation, in addition to an impact on the CO_2_ assimilation capacity to produce more adenosine triphosphate (ATP), which is important for photoreactions, under low temperatures. In this review, some debate aspects related to AM symbiosis and photosynthesis performances will be discussed, with a specific focus on nitrogen acquisition—mediated by AM fungi. 

## 2. AM Symbiosis-Mediated Nitrogen Acquisition in Plant

### 2.1. Nitrogen Use in Agricultural Practices and Biological Significance in the Plant Life

In the twentieth century, agronomic and plant nutrition practices profoundly changed with a widespread use of fertilizers and advances in breeding techniques for high-yielding crops production, known as the ‘Green Revolution’ phenomenon [21,22]. Thanks to the (bio)technological and scientific advances over this period, new crop varieties, together with synthesis of new pesticides, herbicides and inorganic fertilizers, allowed unprecedented yield increase and agricultural modernization [23]. With the enhancing in above ground weight, an increased photosynthate allocation was obtained, leading to a more efficient photosynthesis use. The crops developed during the Green Revolution were domesticated plants selected specifically to respond to fertilizers and produce an increased amount of grain per acre planted. Among fertilizers, the chemically synthesized nitrogen (N) was the main player, considering that its production via the Haber-Bosch industrial process accounted for about 2% of world’s annual energy output [24]. This massive production posed risks for environment and human health and once applied lead to soil quality loss [25,26]. Considering the ongoing climate changing and the consequent predicted stress scenarios that includes novel plant pathogens, intense abiotic stresses (e.g., drought and salinity) and low availability of organic N and phosphorus fertilizers, novel environmental friendly management options (e.g., smart-climate agriculture, including crop diversification and the use of beneficial root-associated microorganisms) are under scrutiny [26,27]. In detail, the final goal of research and industry efforts, would provide an improved N use by crops. The so called N use efficiency (NUE), is commonly defined as the grain or biomass crops yield per unit of N in the soil and its improvement is referred to three major targets: plant breeding, alternative and sustainable agronomic practices and exploiting beneficial effects of microbes inhabiting soil and plant tissues [18] and references therein. Despite the difficulties in dissecting the ecosystem services of beneficial microbes living together in a complex system, thanks to the Next Generation Sequences (NGS) approaches, a huge diversity of microbial communities and their putative roles in crop adaptation strategies and mineral nutrition, (including N cycle) were discovered [28,29,30,31,32].

Plants take up their N predominantly through the roots in different forms, such as nitrate (NO_3_, mobile in soil and mostly used by plants in arable lands), ammonium (NH_4_^+^, form much less mobile in soil and preferred in acidic and anaerobic conditions as for rice plants) or amino acids. The root N uptake can happen directly by means of plant transporters [33,34] and/or indirectly by beneficial microbes associations (e.g., AM fungi) [7,8,35,36]. 

During the vegetative seasons, roots, shoots and young leaves are sink organs for nitrogen, while in the later growth stages (usually after the flowering phase) a re-mobilization of N takes place and both leaves and roots become source organs of amino acids [37]. In general, since the leaves are the main site with nitrate reductase activity in plants, it is well known that plants with low N content appear suffering with smaller and lower number of leaves and premature senescence with respect to those fertilized [38,39]. Additionally, N availability also affects the plant hydraulics; for example, sunflower plants grown in substrate with low N content showed hydraulic conductance impairment, but, once supplied with nitrate fertilization, the hydraulic conductance quickly recovered [40]. Although further studies are needed to deepen this aspect, the conductance increase was likely due to the activation of aquaporin channels mediated by nitrate application [40,41].

Looking at photosynthesis, a strong correlation between N and photosynthetic performances have been reported since the 80 s [42], this because N is one of the main component of chlorophyll, photosynthetic-related enzymes, photosystem proteins and other proteins localized in the chloroplasts membranes. In this line, a direct relation between N content, CO_2_ assimilation rate, chlorophyll content and Rubisco activity have been previously reported in [29,36] and references therein. For these reasons, since the assimilation rate has been measured for many plant species, by combining nitrogen content and leaf dry mass, it is possible to predict, by means of model applications, the photosynthetic capacity of natural ecosystems [43,44,45].

In the optic of sustainable crop management to improve NUE, over the last decades many researches have been performed using transgenic plants (GMOs) or agronomic techniques with limited translational success as a consequence of the strong restrictions and public concerns of GMOs in many countries [6]. For these reasons, in the last few years several researchers focus their attention to study the roles played by beneficial microbes on plant N acquisition [46]. It is well known that microbes carry out essential reactions to convert the different forms of N, thus contributing to the N cycle, although most of the reports present in literature refer to N_2_-fixing bacteria and archaea [47]. More recently the impact of AM symbioses in mediating N uptake to its host become increasingly relevant, as further detailed in the section below.

### 2.2. AM-Mediated Effects in Soil N-Cycling and Plant Acquisition

Nowadays it is well recognized that AM symbioses are able to provide many ecosystem services to their hosts, including nutrients transfer from the soil (e.g., P, zinc, copper), although less importance to N cycling and acquisition have been imputed to AMF [26].

However, AM fungi may affect directly or indirectly N-cycling processes in the soil. Firstly, AMF leads to modifications of soil aggregates and aeration that in turn could influence nitrification/denitrification processes and reduce leaches of inorganic N [8,48]. Moreover, their presence could also affect soil pH and consequently the N availability for the surrounding plants and microbes [49]. In terms of microbial structure and diversity, it is well established that AM root colonization could result in shifting the microbial community in the rhizosphere and in the surrounding bulk soil by means of both fungal and AM-mediated root exudates. This can favor the recruiting of some bacteria genus (e.g., *Azospirillum*, *Pseudomonas*) influencing directly or indirectly soil N-cycling processes [50]. 

**Figure 1:**
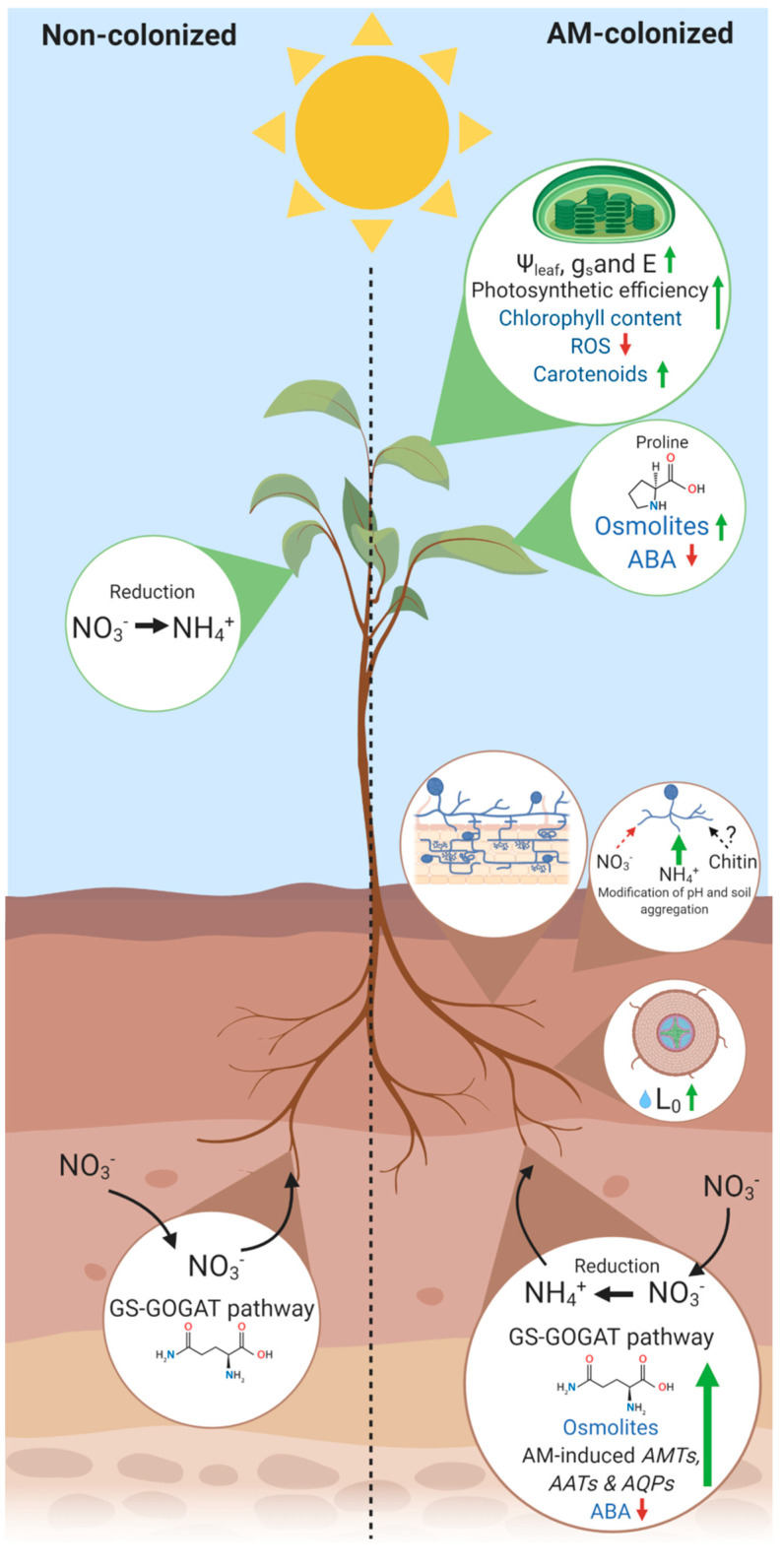
Schematic overview of some biochemical responses to drought, nitrogen uptake, translocation and reduction in AM-colonized and non-colonized plants. The most relevant aspect related to plant nitrogen uptake is the site where nitrate (NO_3_^−^) is reduced to ammonium (NH_4_^+^): when an AM symbiont is present, the reduction takes predominantly place in the roots, while without the AM symbiont it mainly happens in leaves. Glutamine synthetase-glutamate synthase (GS-GOGAT) pathway is involved in the transformation of NH_4_ ions into amino acids and results more active when AM fungi are present. Similarly, the root hydraulic conductivity (L_0_), the photosynthetic activity, the main physiological and biochemical parameters are enhanced in the AM-colonized plants. Blue words represent biochemical responses that occur when the AM-colonized plant is under drought and which are significantly different from non-colonized plant. Green arrows indicate an increase in content/rates whereas red arrows represent a decrease in content/rates respect to non-colonized plants. Ψ_leaf_: leaf water potential; g_s_: stomatal conductance; E: transpiration rate; ROS: reactive oxygen species; ABA: abscisic acid.

On the fungal side, AM fungi are able to actively uptake both NO_3_^−^ and NH_4_^+^ forms, but since the reduction of NO_3_^−^ to NH_4_^+^ is an energy-requiring process, AM fungi prefer to take up the NH_4_^+^ form. They encode for both ammonium transporter (AMT) genes and NO_3_^−^ transporter (NT) gene(s) [51]. Additionally, as revealed by molecular and ^13^C and ^15^N labelling experiments, AMF are capable of taking up and transferring a huge amount of organic N forms—for example, free amino acids (e.g., glutamine, aspartic acid, arginine, proline) or small peptides to their hosts [52,53,54]. Once N has been transferred into the fungal cytoplasm, it is translocated into the intraradical hyphae via vacuole and the NH_4_^+^ form is released in the apoplastic compartment [55,56,57,58]. The latter is thus assimilated and transformed into amino acids, mainly by the glutamine synthetase-glutamate synthase (GS-GOGAT) pathway [58,59]. In the case of NO_3_^−^ uptake by AM fungi, it is subsequently converted by several enzymatic reactions in NH_4_^+^. As cited before, in non-colonized plants, NO_3_^−^ reduction mainly occur in the leaves, while in AM plants it mainly happens in the roots (Figure 1) [60]. In addition, it has been also demonstrated that GS and GOGAT activities were significantly higher in AM-colonized plants with respect to the non-colonized ones [42,43], further underlying the considerable roles that AM symbiosis plays for N assimilation in their plant hosts. More recently, it has been reported that chitin could represent a significant N source for the AM fungi. Chitin, together with cellulose, represent the most abundant polymers in nature, with high content in N and largely present in soil micro-, meso- and macrofauna [41,61]. Bulkovskà and colleagues [62], used ^15^N-labelling technique demonstrating that a large fraction of organic N from chitin has been transferred to the colonized plants in few weeks. Interestingly, genes encoding for the chitin monomer N-acetylglucosamine transporters and metabolism have been documented in *Rhizophagus irregularis*. These findings opened new questions about the potential AM fungi-chitinolytic capacity and the consequent N uptake that still remain to be addressed [26,63].

On the plant side, the final step of AM-mediated nutrients transfer to the host occurs in the periarbuscular membrane, probably by transmembrane transporters that allow their delivery into the cortical cells [64]. It is not yet clear how the plants take up the ammonium released by the AM fungi, although several AM-induced plant ammonium transporters (AMTs) and an aquaporin potentially involved in ammonium uptake have been discovered in many plant species (e.g., *Lotus japonicus*, tomato, *Sorgum bicolor*) [65,66,67]. For example, the first plant AMT gene (*LjAMT2;2*) activated during AM symbiosis was characterized in *L. japonicus* and it is able to transport the NH_3_ molecules as revealed by the *Xenopus laevis* oocytes assay [65]. Looking at NO_3_^−^ transporters, three families have been characterized in plant (NRT1/NPF, NRT2 and NRT3). The AM-induced NO_3_^−^ transporters belonging to the NRT1/NPF and NRT2 families have been identified in several plants [68,69,70,71]. However, the role(s) of these AM-induced NRTs needs yet to be elucidated using functional genomics and subcellular localization approaches. Additionally, as mentioned before, high levels of several amino acids have been reported in mycorrhizal roots, suggesting an active amino acids transfer by AMF to their colonized hosts. In this line, three members belonging to the amino acid permease (AAP) family have been found upregulated only in AM samples of *L. japonicus* roots [70,72]. Interestingly, the *LjLHT1.2*, an amino acid transporter (AAT) gene, encoding for a Lysine-Histidine-Transporter (LHT), was found strongly upregulated in AM cells and the heterologous expression assay confirmed its amino acid transporter competence [53].

In summary, the AMF mycelium represents a sizable N sink for itself and the host plant with consequent competition between the symbiotic partners for N resources when subjected to N-limited conditions. This stress could lead to a shift in the relationship from mutualistic up to parasitism under a severe N limited conditions [73]. Conversely, excessive N supply could decline AM fungal colonization in the roots [74,75], suggesting that N content in soil could also strongly affects the functioning of AM symbiosis as well as the root colonization rate. On the other hand, only when N demand by the fungus has been satisfied do the AMF responses become positive for the plant [76]. 

In the future, a promising strategy to improve plant N nutrition could involve both bacteria and AM fungi, favoring the establishment of so-called tripartite associations [77]. In some cases, it has been found AMF associate with other microbes (mostly with bacteria) with beneficial or detrimental effects to AMF, although such relationships are not well characterized yet [78,79]. These interactions seem to involve fungi exudate products that are able to attract and recruit bacteria that, in turn, facilitate the fungal access to the nutrients. An example has been reported for the phosphate present in soil that was solubilized by some bacteria, thus improving both fungal and plant P nutrition [80]. In this line, a recent report showed that an AMF inoculum combined with a microbial consortium isolated from non-fertilized soils, leads to N uptake improvement in *Brachypodium dystachion* [81]. In addition, several plant-associated fungi, including AMF, are often colonized by endosymbiont (e.g., diazotrophs) that can provide additional N to the fungus, thus ameliorating plant N acquisition and physiological performances, particularly under poor fertilized soil environments [77,82,83]. Recently, de Novais and colleagues [84] demonstrated that the wide network of extraradical mycelium (ERM) in the soil can facilitate translocation and associations of beneficial bacteria (e.g., nitrogen-fixing rhizobia) and fungi to the colonized-plants, as also observed in AM-colonized fenugreek plants under water deficit conditions [79]. Interestingly, it has been recently reported that AM symbiosis can modify the physiology and the environment of the host plant leading to an enhanced nutrient uptake, without a direct phosphorous (P) contribution through the fungal hyphae. It has been suggested that P probably increased in colonized plants for a change in the composition of soil microbial community [14]. 

These findings further highlight the great ecological importance of AM fungi in natural- and agro-ecosystems. In fact, the above cited ERM network, can connect roots of different plant species enabling nutrient (N and P in particular) transfer from one plant (donor) to another (receiver) depending on the biomass strength, functioning as an unique super-organism [85,86]. 

## 3. Linking Root-Colonization by AM Fungi to Plant Water Relations, Biochemical and Photosynthetic Performances

In the optic of sustainable crop management, the improvement of water use efficiency (WUE) is another important target to maintain crop yield and reduce water consumption. Since under drought the uptake of nutrients by roots and their translocation to aerial parts is impaired by low soil water availability, the AM symbiosis has been widely utilized to improve plant performance under drought conditions, both in natural- and agro-ecosystems [87,88,89,90,91,92]. It has been shown that AMF symbiosis increases plant water content by the mycorrhizal root system through extra-radical phase [93,94]. Indeed, AM hyphae can obtain water resources from the soil both by exploring micropores not accessible to plant roots and by affecting soil structure stability [95,96]. As a consequence, the resistance to water movement through the soil, also in areas outside of the root zone, is reduced and plant water uptake results are facilitated [37,89,97] (Figure 1). A further beneficial effect on plant water relations may derive from an improved AMF-mediated P uptake [98] or, as suggested by Hoeksema et al. [99], from different N:P ratios in mycorrhizal plants compared to non-inoculated ones. More recently, Quiroga et al. [100] reported that N fertilization with either ammonium or urea increased net photosynthesis (A_N_) and stomatal conductance (g_s_) in plants maintained under well-watered conditions, with an increase in A_N_ under high NH_4_^+^ supply in AM-colonized plants with respect to non-AM plants. However, fourteen days after drought stress imposition, these parameters decreased in AM plants fertilized with high N doses. These results have been correlated to a differential regulation of aquaporins both regulated by N status and AM symbiosis, suggesting a possible role in the AM-mediated plant N homeostasis that requires further analyses. 

In addition to these direct mechanisms which affect the availability of water and nutrient uptake, several molecular and biochemical mechanisms have been proposed to explain the enhanced performance upon drought mediated by AM symbiosis in the host plant. At root level, the establishment of the AM symbiosis induces extensive alterations in root morphology and physiology, and these changes are likely to be controlled by specific gene expression pattern in the host plant [101]. In addition to nitrate transporters and plant glutathione-S-transferases (GSTs), several studies have shown the up-regulation of some aquaporin genes induced by mycorrhizal colonization [66,102,103,104]. Aquaporins are ubiquitous membrane proteins involved in the maintenance of plant water homeostasis facilitating the flux of water and small solutes [105,106,107]; however, the role of aquaporins in AM symbiosis may be not only restricted to regulate plant water status. Among these roles, AM-regulated aquaporins may be involved in the plant mobilization of N forms (see Section 2.2), such as ammonium and urea [100]. In addition, in a study on AM symbiosis in maize plants under drought, Bárzana et al. [108] have shown the upregulation of some plant aquaporins belonging to the family of the tonoplast intrinsic proteins (TIPs) which can transport H_2_O_2_. Since H_2_O_2_ is a key signalling molecule produced under both biotic and abiotic stresses, this evidence suggests that TIPs could play a key role in the detoxification of excess H_2_O_2_ generated under stress conditions [109]. Regarding the regulation of plant water status, Watts-Williams et al. [110] have suggested that the up-regulation of root aquaporin expression in AMF inoculated plants leads to a significant increment in root hydraulic conductivity (Lo) in several accessions of *Medicago truncatula*. The increment in Lo was positively correlated to the percentage of colonization, thus likely indicating a switching from the cell-to-cell pathway to the apoplastic pathway during the growth of AM colonization [111]. The higher Lo, regulated by specific root aquaporins in AM plants, may explain the maintenance of higher stomatal conductance (g_s_), transpiration rate, relative water content and water potential (Ψ_w_) observed in mycorrhizal plants at low soil water content compared to non-mycorrhizal plants [112,113,114,115,116,117] (Figure 1). The higher foliar water status characters observed in AM plants under drought were also associated with a lower concentration of abscisic acid (ABA) in roots, xylem sap and leaves [114,118]. Since the hormone ABA increases under drought to prevent excessive water loss, such differences indicate that AM plants perceived the water deficit conditions less than their non-mycorrhizal counterparts [119]. The maintenance of a higher g_s_ in AM plants can be helped not only by the higher water influx from the roots to the shoot, but also by an active osmotic adjustment observed in leaves of AM plants e.g., [117]. The accumulation of the osmolytes depends on the AM-plant interactions and may involve both soluble sugar, proline and inorganic ions, mainly K^+^ and Cl^−^ [118,120,121,122,123,124]. Regarding the release of soluble sugars, this process results increased in leaves of mycorrhizal tomato plants through the upregulation of the genes coding for the vacuolar invertase *TIV1* and the cell wall invertase *LIN6*, which both cleave sucrose into fructose and glucose [16]. This evidence suggests that genes involved in the accumulation of soluble sugars are relevant to how leaves respond to mycorrhizal colonization and further studies will be needed to investigate the regulation of the additional pathways involved in the release of osmolytes during AM symbiosis. Indeed, the net accumulation of osmotically active solutes has a key role in the maintenance cell turgor, which allows important processes such as cellular growth and photosynthetic performances, as recently observed by Mo et al. [125], which reported higher Rubisco activity in AM inoculated watermelon seedlings than non-inoculated ones under drought. These authors reported a considerable reduction in the A_N_ of watermelon seedlings under water limitation conditions, with an alleviation of the negative effect due to the AM symbiosis, in agreement with previous studies [126,127]. Suppression of photosynthesis in drought conditions can be correlated to stomatal limitation and/or non-stomatal/metabolic limitation [128]. Mo et al. [125] suggested that the difference in photosynthetic efficiency between watermelon mycorrhizal and non-mycorrhizal seedlings was probably due to non-stomatal rather than stomatal limitation. These authors reported that although the maximum photochemical efficiency of PSII (Fv/Fm) was negatively affected by water limitation, this variable was significantly higher in the leaves of the mycorrhizal watermelon plants compared with the non-mycorrhizal one. This result, together with the higher Fv/Fm, electron transport rate (ETR), photochemical (qP) and non-photochemical quenching (NPQ) and the lower induction of two genes involved in the process of chlorophyll breakdown (i.e., *PAO* and *PPH* genes) in AM-colonized plants under drought stress compared to non-colonized plants, suggested that root colonization by AM fungi can reduce damages and sustain the efficiency of PSII photochemistry at a relatively high level. RuBisCO activity is correlated with the expression of genes encoding for small (rbcS) and large (rbcL) RuBisCO subunits and depends on the activity of RuBisCO activase (RCA) [129]. Chen et al. [130] reported that the AM fungal colonization of cucumber roots, in addition to leading to an improved CO_2_ assimilation and gas exchange parameters, positively affected activities and gene expression of a range key enzymes in Calvin cycle, such as *RCA*, *FBPase*, *FBPA*, *SBPase*, *rbcS* and *rbcL* genes. The same authors also reported a significantly higher chlorophyll content in AMF-inoculated plants that was accompanied with an increased N status in the roots. Because chlorophyll molecules trap N, the enhanced N uptake related to AM colonization might be correlated to the higher chlorophyll contents in the AM-colonized plants compared to non-mycorrhizal ones [131]. Additionally, the increased chlorophyll contents in AM-colonized plants has been also associated with increased P and Mg uptake [132,133]. Since g_s_ and chlorophyll content both increased in AM-colonized plants, both stomatal and non-stomatal factors have been suggested to be involved in the photosynthesis improvement in cucumber seedlings [130]. However, in addition to opening and closing the stomata, plants may exert control over their gas exchange rates by varying stomata density in new leaves. Chitarra et al. [118] showed that the tomato root colonization by the AM fungus *Rhizophagus intraradices* determined a higher stomatal density, increasing the plant CO_2_ absorption capacity, in agreement with the significantly higher A_N_ measured in these plants both under irrigated and water stress conditions in respect to uncolonized plants, which were also directly correlated with intrinsic WUE values. It has been also suggested that inoculation with multiple mycorrhizal fungi, genetically distant, might induce higher photosynthetic ability and nutrient uptake in AM plants, and consequently lead to enhanced plant biomass, with respect to a single inoculum or to closely related AM fungal species [130]. It has been also reported that AM fungal colonization can enhance salinity tolerance by increasing photosynthetic capacity, water status and K^+^/Na^+^ homeostasis. Upon saline conditions, black locust AM fungal colonization significantly improved the net photosynthetic rate, quantum efficiency of photosystem II photochemistry, as well as the expression of three chloroplastic genes (*RprbcL*, *RppsbA*, and *RppsbD*) with respect to non-mycorrhizal plants [133]. A proteomic analysis on *Phragmites australis* under metal-stressed conditions also showed that photosynthetic changes due to AM colonization mainly involved the up-regulation of transmembrane protein-pigment complexes CP43 (photosystem II) and FNR (ferredoxin-NADP^+^ oxidoreductase related to photosynthetic electron transport) [123]. Interestingly, a meta-analysis on the effect of AM fungi on plant tolerance to salt stress [19] suggested that, in terms of photosynthetic pathway, mycorrhizal C3 plants more positively responded in terms of gas exchange compared to C4 plants. This has been reported also by Li et al. [134], that showed an AM-mediated drought tolerance higher for a C3 species than that for the C4 species under both light and moderate water stress conditions, suggesting that that AM fungi might have an important role in shaping plant community composition, favoring more C3 species than C4 species under drought. The higher photosynthetic efficiency, primarily obtained through increasing gas exchange capacity and the PSII efficiency (Figure 1), may derive also from a lower oxidative damage and higher membrane stability observed in AM plants [95]. In fact, in plants subjected to water limitation, the mycorrhizal colonization can reduce leaf H_2_O_2_ content and lipid peroxidation [135], while increases activities of specific antioxidant enzymes, in particular of some Mn-SOD (superoxide dismutase) isoforms [118,136,137,138]. The overall effects attributed to AM symbiosis to alleviate the deleterious effects of osmotic stresses by elevating the activities of antioxidant enzymes in leaves and shoots have also been confirmed by studies conducted on salinity stress [125,139]. These biochemical observations were recently confirmed on shoots of *S. cannabina* seedlings exposed to salinity stress by qRT-PCR, in which in Ren et al. [140] found increased expression levels of genes related to SOD, catalase (CAT) and glutathione reductase (GR). As a consequence, the AMF-mediated maintenance of redox homeostasis in osmotic stressed plants leads to the protection of the major metabolic pathways, including the biosynthesis of chlorophyll and carotenoids, which contribute to preserving the photosynthetic process under stress conditions [141]. The overall effects attributed to AM symbiosis to alleviate the deleterious effects of osmotic stresses also include the activation of ROS scavenging non–enzymatic pathways, such as polyphenols [142,143]. However, while the accumulation of polyphenols in the roots of mycorrhizal plants has been reported in several studies [128,144], the influence of AM colonization on polyphenol biosynthesis in plant leaves has been poorly investigated [145,146]. Further studies aimed at the molecular and functional characterization of genes related to secondary metabolites in different plant tissues will be required to highlight the role of these molecules in water stress tolerance induced by AM symbiosis. Additionally, considering that the involvement of aquaporins (AQPs) in stomatal conductance, transpiration and photosynthesis has been suggested [147], it could be also interesting to verify if AM symbiosis might systemically affect the regulation of specific AQP isoforms acting in the guard cells and/or subsidiary cells.

## 4. Conclusions

Although several advancements in the knowledge of the role of AM symbiosis in the enhanced plant performance have been done, comprehensive mechanisms must be still investigated using quantitative physiological tools. In recent years, high-throughput plant phenotyping (HTPP) strategies have been widely developed to evaluate the plant performance, including both growth and physiological traits, with the aim to improve the sustainability of agricultural production through the identification of crop genotypes adapted to future climatic conditions. Although several studies have already been conducted on the impact of AM fungal colonization on phenotypical traits, the knowledge on AM-mediated photosynthesis mechanisms and on the role of this fungi in NUE is still patchy and limited information is available on the response of different genotypes to AM symbiosis. For this reason, the integration of the HTPP strategies—which allow us to measure a high number of plant individuals at the same time—in AM symbiosis studies might represent a promising tool to exploit these beneficial root associations for future breeding programs and sustainable agricultural practices.
